# Single-Nucleus Transcriptomics Identifies Stage-Associated Differences in the Olfactory Epithelium During Natural Sex Reversal in *Monopterus albus*

**DOI:** 10.3390/ani16203233

**Published:** 2026-10-14

**Authors:** Yu Wang, Ruoyu Liu, Lili Wang, Fangyuan Zhang, Yilin Yao, Yanlong Song, Zhaoxian Li, Xinhua Chen, Wei Hu, Xuefan Cui

**Affiliations:** 1 State Key Laboratory of Mariculture Breeding, Key Laboratory of Marine Biotechnology of Fujian Province, College of Marine Sciences, Fujian Agriculture and Forestry University, Fuzhou 350002, China; simonedejean@outlook.com (Y.W.); liuruoyu13@mails.ucas.ac.cn (R.L.); 13869770215@163.com (F.Z.); yaoyilin1230@163.com (Y.Y.); chenxinhua@tio.org.cn (X.C.); 2 College of Application of Engineering, Henan University of Science and Technology, Sanmenxia 472000, China; wangliliusm@gmail.com; 3 State Key Laboratory of Breeding Biotechnology and Sustainable Aquaculture, Hubei Hongshan Laboratory, Institute of Hydrobiology, Chinese Academy of Sciences, Wuhan 430072, China; songyanlong@ihb.ac.cn (Y.S.); lizhaoxian@ihb.ac.cn (Z.L.); 4 Key Laboratory of Exploration and Utilization of Aquatic Genetic Resources, Ministry of Education, Key Laboratory of Freshwater Aquatic Genetic Resources, Ministry of Agriculture and Rural Affairs, Shanghai Ocean University, Shanghai 201306, China

**Keywords:** *Monopterus albus*, natural sex reversal, olfactory epithelium, single-nucleus RNA sequencing, olfactory sensory neuron, crypt neuron, neurogenesis, cell–cell communication

## Abstract

The rice field eel (*Monopterus albus*) undergoes natural protogynous sex reversal, but whether peripheral sensory tissues differ across sexual stages remains poorly understood. We used single-nucleus RNA sequencing to characterize the olfactory epithelium (OE) of female, intersex, and male rice field eels. After quality control, 22,959 nuclei were retained and classified into 19 cell populations, including olfactory sensory neurons (OSNs), basal/progenitor, epithelial, mesenchymal, endothelial, and immune populations. OE tissues from five individuals within each sexual stage were pooled prior to nuclei isolation, resulting in one pooled snRNA-Seq library per stage; therefore, between-stage comparisons are interpreted as exploratory and descriptive rather than as independently replicated population-level differences. The relative representation of captured nuclei differed among stages, particularly within the sensory neuronal and basal-cell compartments. Pseudotime analysis placed immature OSNs at relatively early transcriptional states associated with ciliated and microvillous OSN populations, whereas CrOSNs were not continuously connected to the principal iOSN–cOSN/mOSN trajectory. Cell-type-specific analyses identified stage-associated transcriptional differences in basal cells and multiple OSN populations. Predicted cell–cell communication profiles also differed among stages, with male OE showing a larger nominal number of inferred interactions but lower overall predicted interaction strength. Expression patterns of *ompa*, *gng8*, and *ora4* supported the marker-based annotation of major OSN populations within the dataset. These findings provide an exploratory single-nucleus framework for investigating stage-associated variation in the peripheral olfactory system of *M. albus*.

## Simple Summary

The rice field eel (*Monopterus albus*) undergoes natural protogynous sex reversal from female to male through an intermediate intersex stage, but whether its peripheral sensory tissues differ across sexual stages is poorly understood. Given the important roles of olfaction in reproduction and social communication in fishes, we examined the olfactory epithelium of female, intersex, and male rice field eels using single-nucleus RNA sequencing. We identified 19 major cell populations and observed stage-associated differences in the relative nuclear representation and transcriptional profiles of olfactory sensory neurons, basal and progenitor cells, epithelial cells, and immune populations. Immature olfactory sensory neurons showed transcriptional similarities to ciliated and microvillous olfactory sensory neurons, while crypt olfactory sensory neurons displayed a more distinct transcriptional pattern. Predicted cell–cell communication profiles also differed among the three stages. Because tissues from five individuals within each stage were pooled to generate one sequencing library per stage, these between-stage comparisons should be interpreted as exploratory and descriptive. Overall, the study provides an initial cellular framework for investigating olfactory epithelial differences in fishes undergoing natural sex reversal.

## 1. Introduction

Sequential hermaphroditism represents one of the most remarkable forms of adult sexual plasticity in vertebrates. Unlike gonochoristic species, sequentially hermaphroditic fishes can transition from one functional sex to another after sexual maturation, requiring coordinated reorganization of gonadal, endocrine, neural, metabolic, and behavioral systems. The rice field eel *Monopterus albus* is a classical protogynous hermaphroditic teleost that normally develops first as a functional female, passes through an intersex stage containing both ovarian and testicular characteristics, and subsequently becomes a functional male [1,2]. This natural female-to-male transition makes *M. albus* an important vertebrate model for studying the mechanisms underlying sexual plasticity.

Considerable progress has been made in understanding gonadal and molecular changes associated with sex reversal in *M. albus*. Transcriptomic analyses of the brain and gonad have demonstrated extensive stage-dependent regulation of genes and signaling pathways during the female–intersex–male transition [3]. Gonadal transcriptomic studies have further revealed profound reorganization of steroidogenic, germ-cell, and sex-differentiation programs [4]. Epigenetic regulation is also involved, including changes influencing the gonadotropin-responsive regulation of ovarian aromatase *cyp19a1a* [5]. Differential expression of microRNAs and mRNAs provides additional evidence that sex reversal represents a broad regulatory transition rather than a simple morphological replacement of ovarian tissue by testicular tissue [6]. However, most previous work has concentrated on the gonad, brain, and reproductive endocrine system.

Whether peripheral sensory organs show stage-associated differences during natural sex reversal remains largely unexplored. This question is potentially important because fishes use chemical information in feeding, predator avoidance, migration, social interaction, mate recognition, courtship, and reproduction [7,8]. The olfactory epithelium (OE) is the first cellular interface between environmental chemical cues and the nervous system [9]. Changes in reproductive status could therefore be associated with differences in the cellular composition or transcriptional state of the sensory machinery that detects and transmits biologically relevant olfactory information.

Teleost OE is a highly heterogeneous neuroepithelium. In addition to mature and immature olfactory sensory neurons (OSNs), it contains basal stem and progenitor cells, supporting and secretory epithelial populations, stromal and vascular cells, and resident or infiltrating immune populations [7]. Among the neuronal populations, ciliated OSNs (cOSNs) and microvillous OSNs (mOSNs) constitute two major sensory classes with distinct receptor repertoires and intracellular signaling machinery. Additional specialized OSNs include crypt neurons, together with other rare sensory classes, such as kappe neurons in zebrafish [10,11].

The OE is also characterized by lifelong neurogenesis. In teleosts, globose basal cells (GBCs) and other basal/progenitor populations contribute to neuronal maintenance and repair, whereas horizontal basal cells (HBCs) have been extensively characterized as reserve progenitors in mammalian OE, and related basal populations have also been described in fishes [12,13,14]. Because functional equivalence among basal-cell classes across vertebrate taxa is not necessarily complete, the relative nuclear representation and transcriptional states of basal cells, immature neurons, and mature OSNs are used here as descriptive indicators of OE cellular heterogeneity rather than direct measures of regenerative activity.

Crypt olfactory sensory neurons (CrOSNs) are of particular interest in reproductive sensory biology. These rare sensory neurons exhibit distinct morphological and molecular properties, and in zebrafish, are strongly associated with *ora4* expression [15]. Recent functional evidence in zebrafish demonstrated that olfactory GnRH3-associated crypt sensory neurons can transduce sex-pheromone information and contribute to male courtship and reproductive success [16]. Whether comparable relationships exist in *M. albus* is unknown; these findings therefore provide a hypothesis-generating rationale for examining CrOSNs across sexual stages rather than evidence for a conserved mechanism.

Single-cell and single-nucleus transcriptomics provide powerful approaches for resolving such tissue-level heterogeneity and stage-associated variation. Conventional bulk RNA sequencing cannot readily distinguish changes caused by altered cell abundance from transcriptional changes occurring within individual populations. In contrast, single-nucleus RNA sequencing (snRNA-Seq) enables the simultaneous identification of heterogeneous cellular populations and characterization of their transcriptional states. Recent studies have successfully applied single-cell transcriptomics to zebrafish OE [7] and snRNA-Seq to rare GnRH3-associated neuronal populations [17], demonstrating the value of these approaches for investigating olfactory and reproductive neuronal heterogeneity.

In the present study, we constructed an snRNA-Seq atlas of the OE at three stages of natural sex reversal in *M. albus*: female, intersex, and male. We aimed to (1) characterize the major neuronal and non-neuronal populations of the OE; (2) describe stage-associated differences in relative nuclear representation; (3) examine transcriptional relationships among major OSN populations; (4) identify exploratory cell-type-specific transcriptional differences among sexual stages; and (5) characterize stage-associated differences in predicted intercellular communication profiles. We hypothesized that the peripheral OE would exhibit stage-associated cellular and transcriptional differences across natural sex reversal.

## 2. Materials and Methods

### 2.1. Animals and Determination of Sexual Stage

Adult *M. albus* were obtained from Baishazhou Farmer’s Market, Wuhan, China and maintained in a single 500-L holding tank containing tap water at an approximate stocking density of one fish per 10 L of water. Water temperature was approximately 25 ± 2 °C, and water was changed three times daily. Rice field eels at the female, intersex, and male stages had body weights of 40–48 g, 85–88 g, and 101–105 g, respectively, and total lengths of 15–19 cm, 28–29 cm, and 30–36 cm, respectively. Rice field eels were acclimated for a week prior to tissue collection under a 12 h light/12 h dark photoperiod and fed a commercial diet (Aller Aqua, Christiansfeld, Denmark) twice daily. Prior to tissue collection, rice field eels were anesthetized with MS-222 (tricaine methanesulfonate; 70 mg/L; Sigma-Aldrich, St. Louis, MO, USA) for 8 min, followed by euthanasia through spinal cord transection. Tissue samples were subsequently collected for further analysis. OE tissues were collected immediately and snap-frozen in liquid nitrogen pending confirmation of sexual stage and subsequent nuclei isolation. The study was reviewed and approved by the Laboratory Animal Ethics and Welfare Committee, College of Marine Sciences, Fujian Agriculture and Forestry University (approval code: CEREAW202560191; approval date: 20 June 2025).

Sexual stage was initially assessed according to gross gonadal morphology during dissection and subsequently confirmed by gonadal histology, following the established description of natural sex reversal in *M. albus* [1,2]. Gonadal tissues were fixed overnight in Bouin’s solution at 4 °C. The fixed samples were dehydrated in an alcohol gradient, cleared in xylene, and embedded in paraffin. Paraffin sections were cut to a thickness of 5 μm, rehydrated, and stained with hematoxylin and eosin. Images were collected and analyzed using an automatic digital slide scanner (Aperio VERSA 8; Leica Biosystems, Wetzlar, Germany). Histological confirmation identified predominantly ovarian tissue in females, simultaneous ovarian and testicular characteristics in intersex individuals, and predominantly testicular tissue in males. Representative H&E-stained gonadal sections illustrating the female, intersex, and male stages are provided in Figure S1.

Immediately following euthanasia, OE tissues were collected individually and snap-frozen in liquid nitrogen. After histological confirmation of the gonadal stage, OE tissues from five individuals assigned to the same sexual stage were selected and pooled for nuclei isolation. Accordingly, one pooled snRNA-Seq library was generated for each of the female, intersex, and male groups. The five fish contributing to each pool were not independent biological replicates at the library level. Since sexual stage and library identity were completely confounded, comparisons among these libraries were treated as exploratory and descriptive; observed contrasts cannot be disentangled from individual variation or library-specific technical effects.

### 2.2. Cell Nucleus Isolation, Single-Nucleus Library Construction, and Sequencing

To extract nuclei from tissue, approximately 100 mg of pooled OE tissue obtained in total from five individuals of the same histologically confirmed sexual stage was transferred into 2 mL Dounce homogenizer (KIMBLE, Wertheim, Germany, D8938-1SET) containing 1 mL of ice-cold homogenization buffer and incubated on ice for 3–5 min. The homogenization buffer consisted of 250 mM sucrose (Invitrogen, Carlsbad, CA, USA, 15503022), 10 mg/mL bovine serum albumin (BSA; Sigma-Aldrich, A1933), 5 mM MgCl_2_ (Sigma-Aldrich, 63069), 0.12 U/μL RNase inhibitor (Promega, Tokyo, Japan, N2115), and 1× cOmplete Protease Inhibitor Cocktail (Roche, 11697498001). The tissue was gently homogenized with 10 strokes using the Dounce homogenizer. Nuclei release and the presence of tissue debris were monitored microscopically, after which the homogenate was filtered through a 40-μm cell strainer (Falcon™ Cell Strainers, 352340). Nuclei were pelleted at 500× *g* for 5 min at 4 °C and resuspended in buffer B containing 320 mM sucrose, 10 mg/mL BSA, 2 mM magnesium acetate, 3 mM CaCl_2_, 10 mM Tris-HCl, 0.1 mM EDTA, 1 mM DTT, 0.12 U/μL RNase inhibitor, and 1× cOmplete Protease Inhibitor Cocktail [18]. The nuclei suspension was adjusted to approximately 1000 nuclei/μL for library preparation. Single-nucleus RNA-Seq libraries were prepared using the DNBelab C Series High-throughput Single-cell RNA Library Preparation Set V3.0 (MGI, 940-001818-00) according to the manufacturer’s instructions. Qualified libraries were sequenced on the DNBSEQ platform using paired-end 100-bp reads.

### 2.3. Data Processing, Quality Control and Clustering

Raw sequencing data were processed using dnbc4tools (v3.1) according to the manufacturer’s recommended pipeline. Briefly, reads were subjected to quality filtering, barcode and unique molecular identifier (UMI) processing, and alignment to the rice field eel reference genome (GCF_001952655.1_M_albus_1.0). The cell-gene UMI count matrix was used for downstream analyses.

Single-nucleus RNA-Seq data were analyzed using the Seurat R package (v5.5.1) [19]. Low-quality nuclei were excluded using the following dataset-specific criteria: fewer than 200 detected genes, fewer than 200 UMIs, or greater than 5% mitochondrial gene counts. Genes expressed in fewer than three nuclei were also removed. Doublets were identified and removed using the scDblFinder R package (v1.24.0) prior to further analysis. Retained count matrices were normalized using log-normalization (scale factor = 10,000), followed by the identification of highly variable genes (nfeatures = 3000, method = vst). Principal component analysis (PCA) was performed on scaled data, and the top 40 principal components were used for neighbor graph construction (FindNeighbors) and unsupervised clustering (FindClusters, resolution = 0.5). Cell clusters were visualized by Uniform Manifold Approximation and Projection (UMAP) [20]. Harmony was used only to obtain a shared low-dimensional representation for integrated visualization, clustering, and cell-type annotation across the three libraries; because library identity was confounded with sexual stage, this step was not interpreted as correction of independently replicated biological batches.

Harmony-corrected embeddings were used solely for integrated visualization, clustering, and cell-type annotation. Subsequent stage-associated analyses, including pseudotime trajectory inference, differential expression analysis, and cell–cell communication analysis, were performed using the original, uncorrected expression data and are interpreted as exploratory because only one pooled library was available per sexual stage.

### 2.4. Cell-Type Annotation

Cluster-specific marker genes were identified using FindAllMarkers with the Wilcoxon rank-sum test (min.pct = 0.1 and logfc.threshold = 0.25). Cell-type identities were assigned based on a combination of marker expression, cluster topology, and previously established teleost OE markers [7,15]. Because several specialized OE populations in *M. albus* have not been independently validated spatially or at the protein level, these annotations should be regarded as marker-based transcriptomic assignments. The number of nuclei assigned to each annotated population was tallied separately for the female, intersex, and male datasets and interpreted as relative nuclear representation rather than absolute tissue cell abundance.

### 2.5. Pseudotime Trajectory Analysis

Nuclei annotated as iOSNs, cOSNs, mOSNs, and CrOSNs were extracted from the three pooled stage-specific libraries and combined into a subsetted Seurat object. This subset was re-normalized and re-scaled, followed by PCA and UMAP using the same parameters as described in Section 2.3, except that Harmony correction was not applied. Thus, the low-dimensional representation used for pseudotime analysis was generated independently of the Harmony-corrected representation used for integrated clustering and cell-type annotation. Pseudotime reconstruction was performed using the Monocle 3 R package (v1.4.27) [21]. The resulting PCA and UMAP embeddings were transferred to Monocle 3, where trajectories were inferred using the learn_graph function. The trajectory root was defined within the iOSN population exhibiting high *gap43* expression because *gap43* is associated with an immature neuronal state; this root assignment represents a biologically informed analytical assumption rather than direct lineage evidence. Pseudotime for each nucleus was calculated as the geodesic distance to the defined root node along the principal graph. Genes exhibiting trajectory-associated expression patterns were identified using Moran’s I statistics implemented in the graph_test function. Nuclei were ordered along pseudotime and visualized according to both cell identity and sexual stage. Because trajectory inference reconstructs transcriptomic relationships from cross-sectional data rather than directly demonstrating lineage, inferred trajectories were treated as hypothesis-generating [17]. As each library corresponded to a single sexual stage, variation attributable to a particular library may also affect the position of nuclei and topology of inferred trajectories; the analysis cannot separate stage-related patterns from library-specific effects.

### 2.6. Cell-Type-Specific Differential Expression Analysis

To explore cell-type-specific transcriptional patterns associated with sex-reversal stages, gene-expression contrasts were assessed separately in HBCs, GBCs, iOSNs, cOSNs, mOSNs, and CrOSNs. Three comparisons were examined: intersex versus female, female versus male, and intersex versus male. For each comparison, positive avg_log2FC values indicate higher expression in the first-listed group relative to the second-listed group, whereas negative values indicate lower expression. The FindMarkers function in Seurat with the Wilcoxon rank-sum test was applied using a log2 fold-change threshold of 0.5 (logfc.threshold = 0.5). Genes passing this threshold were subsequently ranked by their average log2 fold change (avg_log2FC), and up to 10 genes showing higher and lower expression, respectively, were marked for each comparison. Because each sexual stage was represented by a single pooled library, nuclei within a library do not constitute independent biological replicates. Accordingly, *p* values and adjusted *p* values generated from nucleus-level comparisons were not used to establish statistical significance or population-level differential expression. These analyses were used solely to identify exploratory, stage-associated expression patterns in the sampled libraries.

### 2.7. Cell–Cell Communication Analysis

Intercellular communication profiles were inferred independently for the female, intersex, and male OE datasets using CellChat (v2.1.2). As a species-specific ligand–receptor database is unavailable for *M. albus*, CellChatDB.human included in CellChat (v2.1.2) was used as an exploratory reference. *M. albus* genes were matched to their human counterparts on the basis of standardized gene symbols. For one-to-many mappings, the expression counts of a single *M. albus* gene were assigned to each corresponding human gene. For many-to-one mappings, expression counts from multiple *M. albus* genes corresponding to the same human gene were summed. This process yielded an expression matrix containing 10,556 unique human gene symbols after the described consolidation for downstream CellChat analysis. Given the cross-species nature of the analysis, the inferred interactions were interpreted as putative conserved cell–cell communication relationships. Cell–cell communication was then inferred using the human ligand–receptor interactions implemented in CellChat. Ligand–receptor pairs for which both the ligand and receptor were represented among the mapped genes were retained, resulting in 358 ligand–receptor pairs for subsequent CellChat analysis. Communication probabilities were calculated using computeCommunProb, and pathway-level probabilities were inferred using computeCommunProbPathway. Pairwise interaction profiles between annotated cell types were visualized using circle plots, heatmaps, and dot plots. Because ligand–receptor relationships were inferred from a human-based database, predicted interactions should be regarded as exploratory candidates rather than experimentally validated interactions in *M. albus*.

Given the evolutionary distance between humans and teleosts and the incomplete ligand–receptor annotation of the rice field eel genome, cross-species mapping may introduce false-positive or biologically mismatched interactions. Accordingly, interaction counts, strengths, and pathway assignments are treated as nominal computational predictions and interpreted qualitatively rather than as direct evidence of in vivo signaling.

## 3. Results

### 3.1. snRNA-Seq Identifies Stage-Associated Differences in the Olfactory Epithelial Cellular Landscape

**Figure 1:**
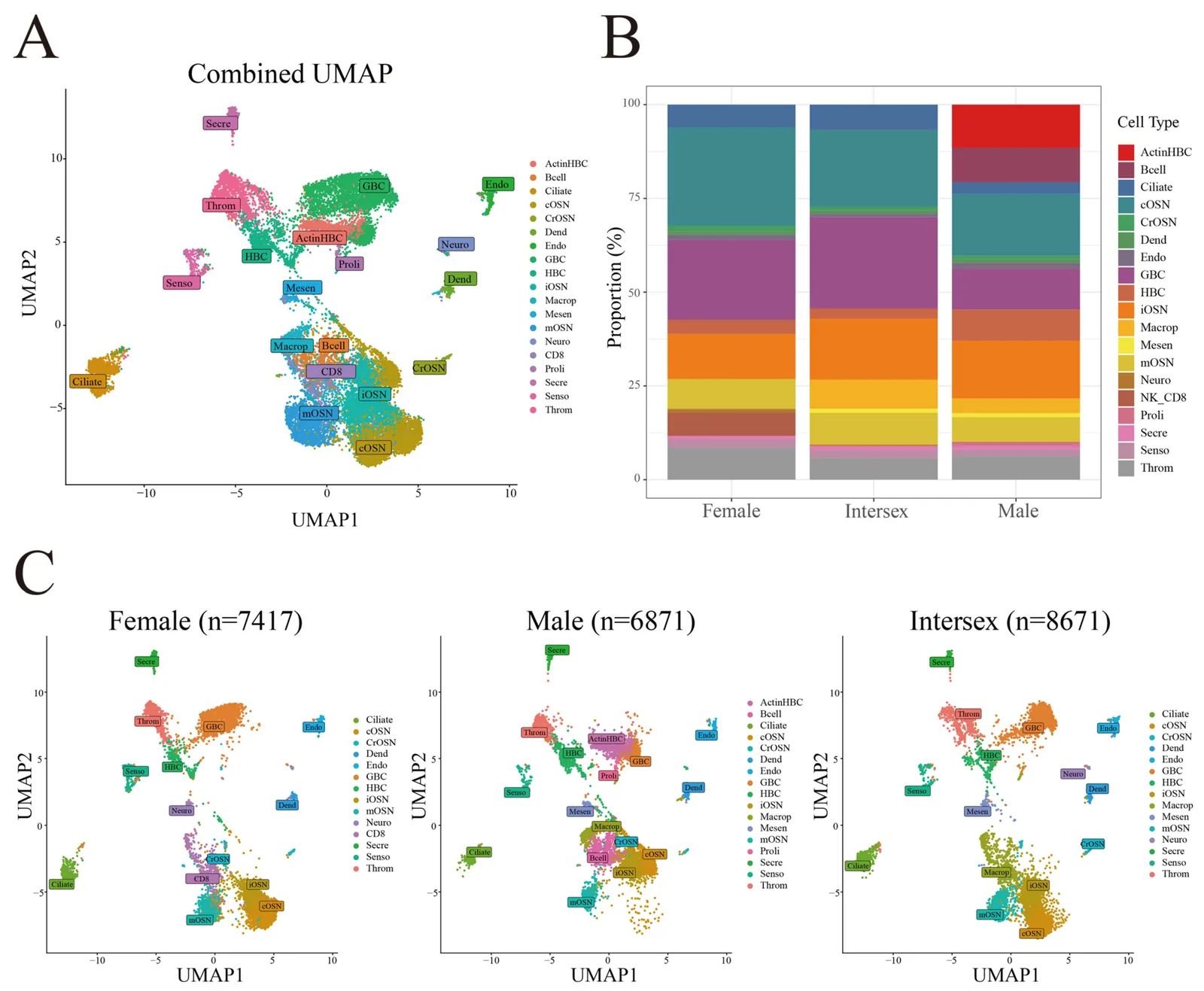
Single-nucleus transcriptomic atlas of the olfactory epithelium across female, intersex, and male *Monopterus albus*. (**A**) Integrated UMAP visualization showing 19 annotated OE populations. Each point represents one nucleus and colors indicate transcriptomic cell identity. (**B**) Relative nuclear representation of annotated populations in female, intersex, and male OE. (**C**) Stage-separated UMAPs showing female (n = 7417 nuclei), intersex (n = 8671), and male (n = 6871) OE. A total of 22,959 nuclei were retained after quality control. Abbreviations: ActinHBCs, activated horizontal basal cells; Bcell, B cells; Ciliate, multiciliated epithelial cells; cOSNs, ciliated olfactory sensory neurons; CrOSNs, crypt olfactory sensory neurons; Dend, dendritic cells; Endo, endothelial cells; GBC, globose basal cells; HBCs, horizontal basal cells; iOSNs, immature olfactory sensory neurons; mOSNs, microvillous olfactory sensory neurons; Macrop, macrophages; Mesen, mesenchymal cells; Neuro, neuroendocrine-like sensory cells; CD8, CD8+ T cells; Proli, proliferating neuronal progenitors; Secre, secretory epithelial cells; Senso, sensory epithelial cells; Throm, thrombocytes. Relative nuclear representation is descriptive because one pooled library was generated per stage.

Single-nucleus RNA sequencing of olfactory epithelium tissue was performed using the DNBelab C4 platform. The estimated numbers of nuclei were 8303, 9791 and 7732, with mean reads per cell of 116,132, 88,038, and 114,656 for the female, intersex, and male libraries, respectively (Table S1). After quality-control filtering, 22,959 nuclei were retained, including 7417 nuclei from female, 8671 from intersex, and 6871 from male OE (Table S2). Integrated analysis classified the nuclei from all three sexual groups into 19 clusters, which were visualized using UMAP (Figure 1A). These clusters were annotated into 19 marker-defined cell types based on cell-type-associated genes and published teleost OE markers: activated horizontal basal cells (ActinHBC; *col17a1b*/*ptprk*), B cells (Bcell; *pou2af1*/*pax5*), multiciliated epithelial cells (Ciliate; *cfap*- and *ccdc*-family genes), ciliated olfactory sensory neurons (cOSNs; *ompa*/*ompb*/*cnga2b*), crypt olfactory sensory neurons (CrOSNs; *ora4*), dendritic cells (*Dend*; *il12bb*/*illr4*/*pik3r5*), endothelial cells (Endo; *fli1*/*tie1*), globose basal cells (GBCs; *msi1b*/*nr2e1*), horizontal basal cells (HBCs; *tp63*), immature olfactory sensory neurons (iOSNs; *gap43*/*stmn1*), microvillous olfactory sensory neurons (mOSNs; *gucy1b2*/*v2r*-family genes), macrophages (Macrop; *lyn*/*runx3*), mesenchymal cells (Mesen; *podn*/*twist1b*), neuroendocrine-like sensory cells (Neuro; *chga*/*hdc*), CD8+ T cells (CD8; *tox2*/*il12rb1*/*il23r*), proliferating neuronal progenitors (Proli; *ntf4*/*anos1*), secretory epithelial cells (Secre; *foxa3*/*fcgbp*/*spdef*), sensory epithelial cells (Senso; *prom2*), and thrombocytes (Throm; *itga2b*) [7,15]. These identities represent transcriptomic annotations and require additional spatial or protein-level validation for definitive cell-type assignment in *M. albus*. Marker genes used for annotation were selected based on established teleost OE literature [7,15] and are summarized in Supplementary Table S3.

The major olfactory sensory populations comprised cOSNs, mOSNs, iOSNs, and CrOSNs. Basal and progenitor populations included GBCs, HBCs, ActinHBCs, and Proli cells. Additional populations included multiciliated, secretory, and sensory epithelial cells, neuroendocrine-like sensory cells, macrophages, dendritic cells, B cells, CD8+ T cells, thrombocytes, mesenchymal cells, and endothelial cells.

The relative nuclear representation of annotated cell types showed apparent differences among the three sexual stages (Figure 1B and Table S2). cOSNs represented a major sensory neuronal population in all three datasets and showed lower relative representation of captured nuclei in the intersex and male datasets than in the female dataset. GBC nuclei were relatively prominent in the female and intersex datasets, whereas HBC and ActinHBC nuclei showed greater relative representation in the male dataset. B-cell nuclei were also more prominent in the male dataset. Stage-separated UMAP visualization showed preservation of the major OE cellular architecture across all three stages together with descriptive differences in the representation of individual populations (Figure 1C). Because each stage was represented by one pooled library, these observations should not be interpreted as statistically validated changes in absolute cell abundance.

### 3.2. Major OSN Populations Occupy Distinct Inferred Transcriptional States

**Figure 2:**
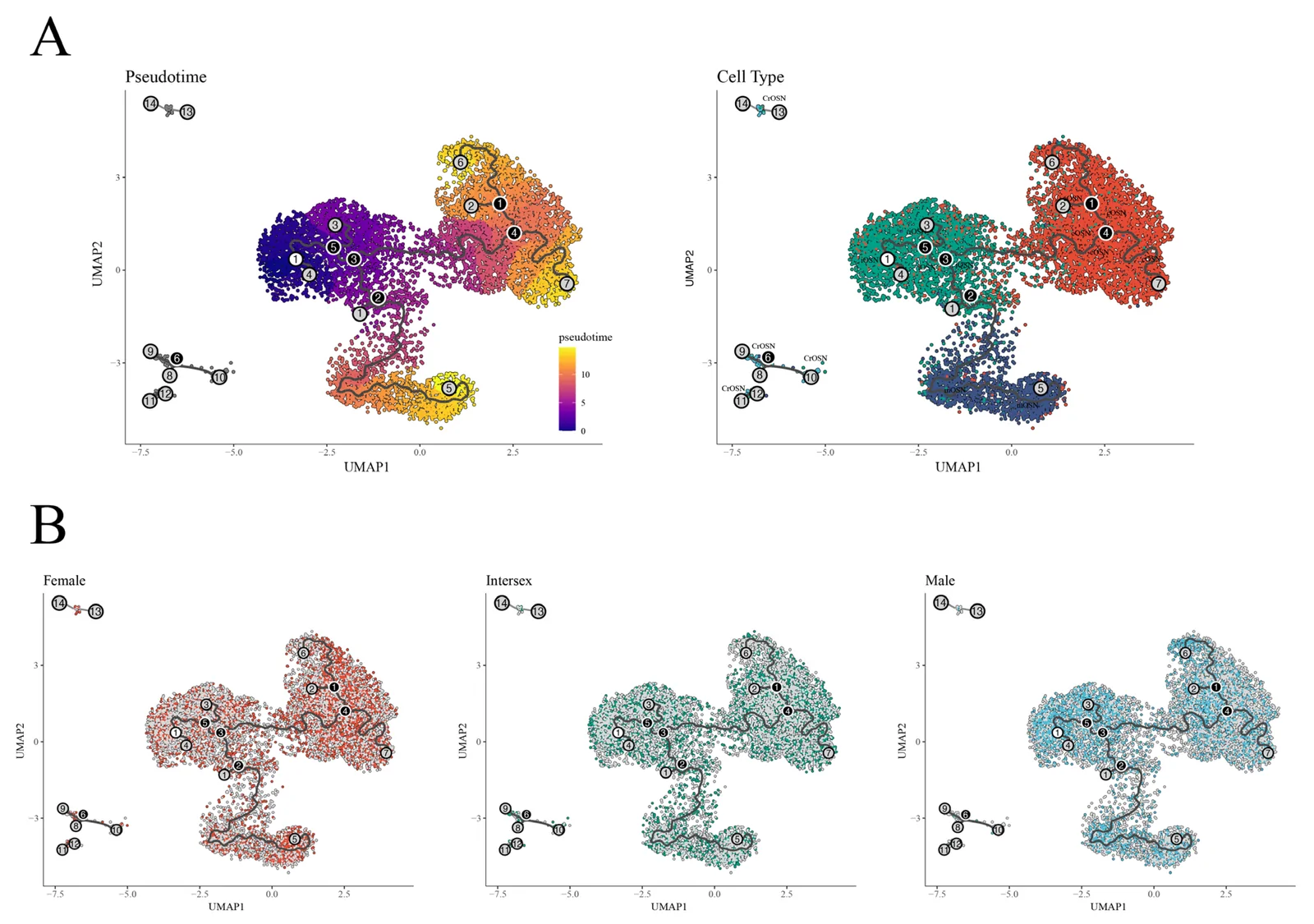
Pseudotime analysis shows distinct inferred transcriptional states of olfactory sensory neurons. (**A**) Inferred trajectory of iOSNs, cOSNs, mOSNs, and CrOSNs. Cells are colored according to pseudotime or cell identity. iOSNs occupy relatively early transcriptional states associated with the principal trajectory containing cOSNs and mOSNs, whereas CrOSNs occupy a relatively distinct region. (**B**) Distribution of female-, intersex-, and male-derived nuclei across the inferred trajectory. Pseudotime represents transcriptomic relationships in cross-sectional data and does not constitute direct lineage evidence. Numbered circles indicate nodes in the inferred Monocle 3 trajectory graph: black circles denote branch points, light-gray circles denote leaf (terminal) nodes, and white circles denote the selected root node. Numbers are reference labels assigned separately within each node category; they do not represent cell counts, pseudotime values, or experimentally established differentiation stages.

Pseudotime trajectory analysis was performed on iOSNs, cOSNs, mOSNs, and CrOSNs (Figure 2). iOSNs were concentrated toward relatively early positions along the principal inferred trajectory, whereas cOSNs and mOSNs occupied later positions. This pattern is consistent with, but does not establish, a transcriptional relationship between immature neuronal states and differentiated ciliated or microvillous sensory populations.

In contrast, CrOSNs occupied a relatively distinct portion of the reconstructed landscape and were not continuously connected to the principal iOSN–cOSN/mOSN trajectory. The present analysis therefore did not resolve a transcriptional trajectory linking CrOSNs to the other major OSN populations. Female-, intersex-, and male-derived nuclei showed different descriptive distributions across portions of the inferred trajectory. These cross-sectional patterns do not establish lineage, temporal progression, or stage-specific differentiation rates.

### 3.3. Natural Sex Reversal Is Associated with Cell-Type-Specific Transcriptional Differences

**Figure 3:**
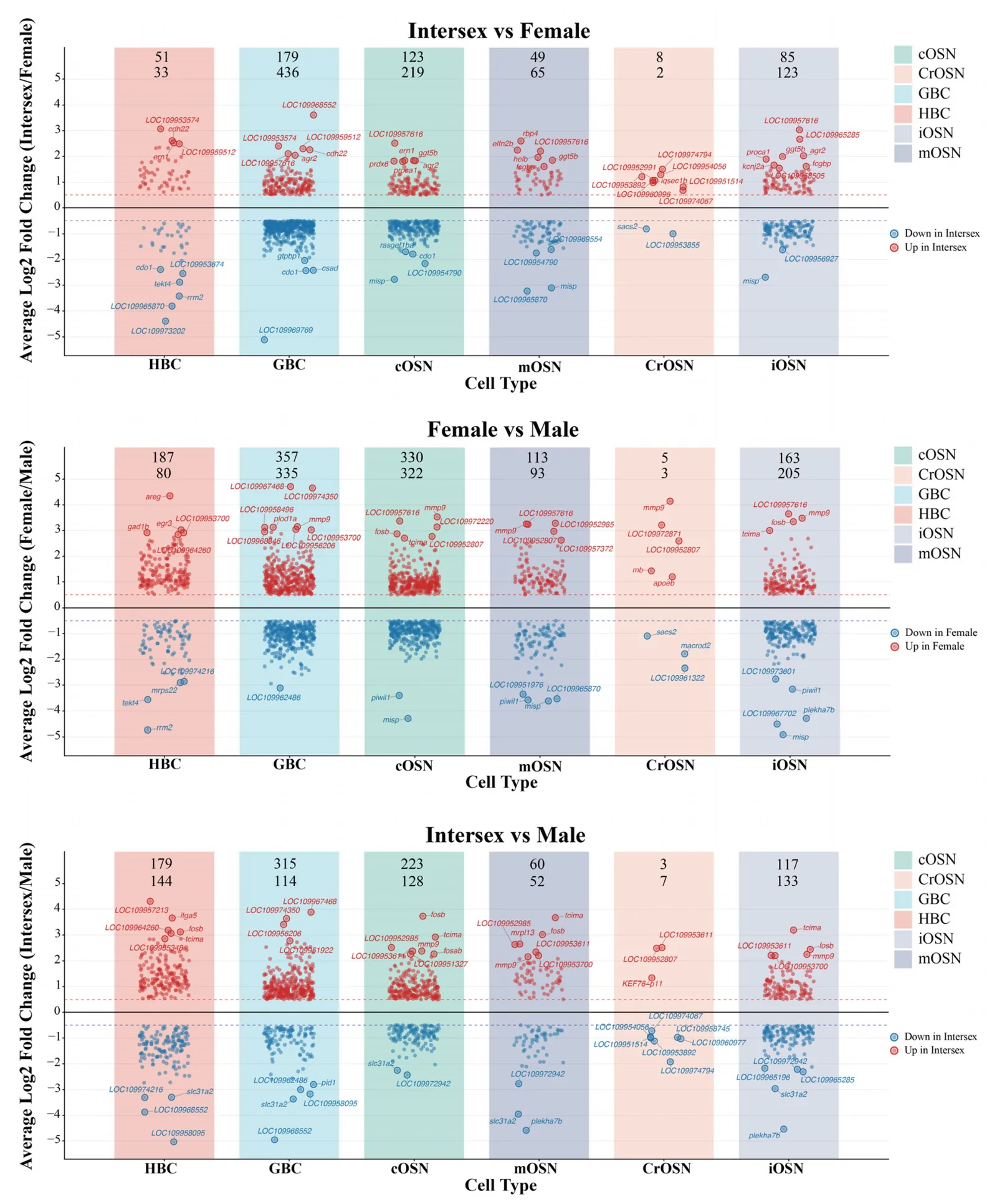
Exploratory cell-type-specific expression patterns across sex-reversal stages. (**Top**) Intersex versus female comparison (*y*-axis: log2[Intersex/Female]) in HBCs, GBCs, iOSNs, cOSNs, mOSNs, and CrOSNs. Red dots indicate higher expression and blue dots indicate lower expression in the Intersex library relative to the female library. (**Middle**) Female versus male comparison (*y*-axis: log2[Female/Male]). Red dots indicate higher expression and blue dots indicate lower expression in the female library relative to the male library. (**Bottom**) Intersex versus male comparison (*y*-axis: log2[Intersex/Male]). Red dots indicate higher expression and blue dots indicate lower expression in the intersex library relative to the male library. Panel headings identify the groups being compared, whereas the y-axis ratios define the direction of the log2 fold change. Genes are displayed according to average log2 fold change. The two numbers shown above each cell-type box indicate the numbers of genes displaying higher or lower expression in the indicated comparison: the upper number corresponds to the number of genes represented by red dots (higher expression), and the lower number corresponds to the number represented by blue dots (lower expression). Because each sexual stage was represented by a single pooled library, the displayed expression contrasts are descriptive and do not represent independently replicated tests of population-level differential expression. LOC or accession identifiers are retained for loci that could not be reliably assigned standardized gene symbols in the current *M. albus* genome annotation. The red and blue horizontal dashed lines indicate avg_log2FC thresholds of +0.5 and −0.5, respectively, used to screen genes for exploratory expression comparisons. The solid horizontal line marks avg_log2FC = 0. These thresholds do not denote statistical significance.

Exploratory gene-expression contrasts were examined within HBCs, GBCs, iOSNs, cOSNs, mOSNs, and CrOSNs (Figure 3). In the figure, positive log2 fold-change values correspond to higher expression in intersex relative to female (top), female relative to male (middle), and intersex relative to male (bottom). In the intersex-versus-female comparison, genes including *cdh22*, *agr2*, *prdx6*, *ggt5b*, *elfn2b*, *rbp4*, *kcnj2a*, and *fcgbp* showed higher expression in one or more examined cell types in the intersex dataset, whereas *misp*, *sacs2*, *cdo1*, *rasgef1ba*, *gtpbp1*, *csad*, and *tekt4* showed lower expression. Because only one pooled library represented each stage, these contrasts were used to identify candidate stage-associated expression patterns and not to infer independently replicated population-level differential expression.

In the female-versus-male comparison, genes including *areg*, egr3, *gad1b*, *plod1a*, *mmp9*, *fosb*, *tcima*, *mb*, and *apoeb* showed lower expression in the male dataset (i.e., higher expression in the female dataset) within one or more cell populations, whereas *mrps22*, *tekt4*, *piwil1*, *misp*, *sacs2*, *macrod2*, and *plekha7b* showed higher expression in the male dataset. In the intersex-versus-male comparison, *itga5*, *fosb*, *tcima*, *mmp9*, *fosab*, and *mrpl13* showed lower expression in the male dataset (i.e., higher expression in the intersex dataset), whereas *slc31a2*, *plekha7b*, and *pid1* showed higher expression in the male dataset. Several genes, particularly *fosb*, *tcima*, *mmp9*, *slc31a2*, and *plekha7b*, appeared across multiple exploratory comparisons or populations. Because the current *M. albus* genome annotation remains incomplete, a subset of differentially expressed loci could only be represented by LOC/accession identifiers and could not be reliably assigned standardized gene symbols or full gene names.

### 3.4. Predicted Cell–Cell Communication Profiles Differ Across Sex-Reversal Stages

**Figure 4:**
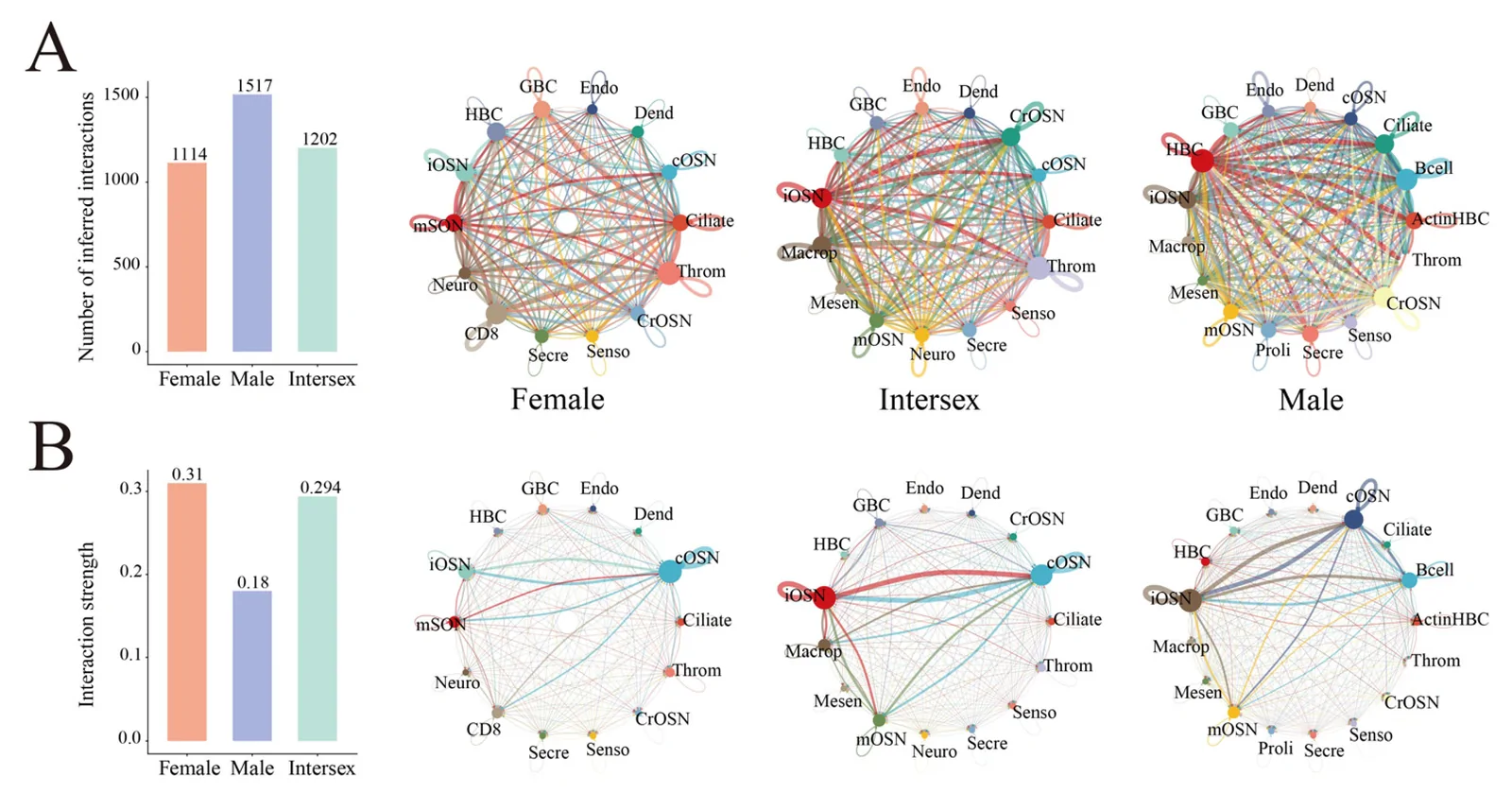
Exploratory prediction of intercellular communication profiles across sexual stages. (**A**) Nominal numbers of predicted cell–cell interactions in female (1114), intersex (1202), and male (1517) OE. (**B**) Overall predicted interaction strengths in female (0.310), intersex (0.294), and male (0.180) OE. All values were computationally inferred using CellChatDB.human and should be interpreted qualitatively rather than as experimentally validated communication events.

Exploratory CellChat analysis identified stage-associated differences in predicted communication profiles (Figure 4). The nominal numbers of inferred ligand–receptor interactions were 1114 in female, 1202 in intersex, and 1517 in male OE, whereas the corresponding overall predicted interaction strengths were 0.310, 0.294, and 0.180, respectively. These values are computational estimates generated using CellChatDB.human and should not be interpreted as directly measured communication events or as quantitatively validated differences among sexual stages.

Outgoing- and incoming-signaling profiles also differed descriptively among datasets. cOSNs remained prominent within the predicted communication networks, whereas iOSNs showed comparatively stronger predicted communication profiles in intersex and male datasets. mOSNs and CrOSNs also occupied different positions within the inferred networks. These patterns should be regarded as candidate signaling relationships for future validation because they depend on cross-species ligand–receptor mapping and one pooled library per stage.

### 3.5. Expression Patterns of Representative Marker Genes Across Major OSN Populations

**Figure 5:**
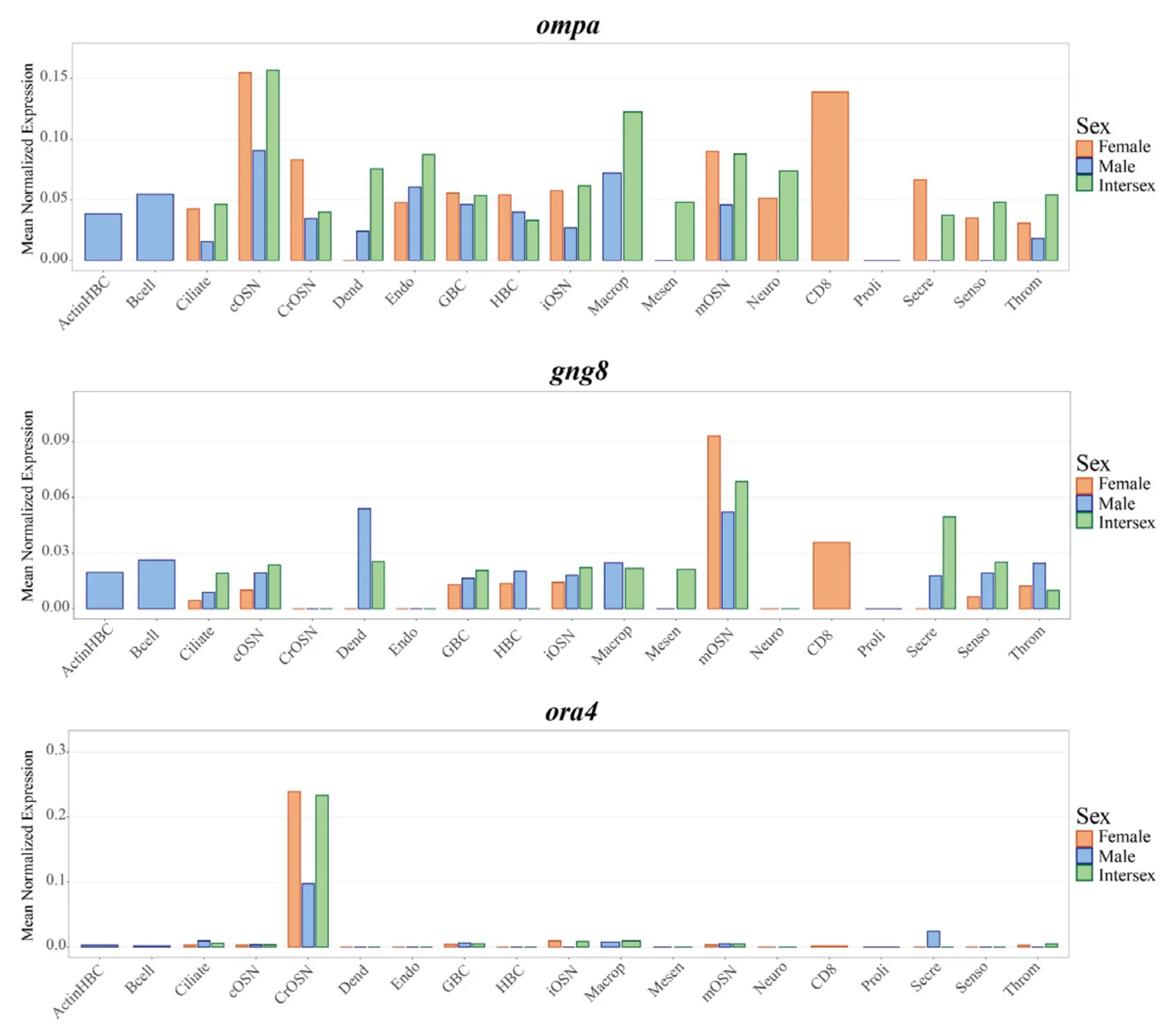
Expression patterns of representative marker genes across major olfactory sensory neuron populations. snRNA-Seq-derived expression patterns of *ompa*, *gng8*, and *ora4* across annotated cell populations and sexual stages are shown. *ompa* displayed its highest expression in cOSNs, *gng8* was enriched in mOSNs, and *ora4* showed strong specificity for CrOSNs. Bars show the mean normalized expression across cells within each cell type in each pooled stage-specific library. No error bars are displayed, because these within-library summaries do not represent variation among independent biological replicates or individual fish. These patterns support marker-based cell annotation within the present snRNA-Seq dataset but do not constitute independent RT-PCR, RT-qPCR, spatial, or protein-level validation.

snRNA-Seq expression patterns were examined for representative OSN-associated genes (Figure 5). Within the present dataset, *ompa* showed its highest expression in cOSNs, *gng8* showed enrichment in mOSNs, and *ora4* showed strong specificity for the CrOSN cluster. These patterns support the marker-based transcriptomic annotation of the major OSN populations within the snRNA-Seq dataset. Because the same dataset was used for clustering, annotation, and visualization, these expression patterns do not constitute independent experimental validation of between-stage differences.

## 4. Discussion

### 4.1. Stage-Associated Differences in the OE Across Natural Sex Reversal

The present study provides a single-nucleus transcriptomic characterization of the *M. albus* OE across female, intersex, and male stages. The principal finding is that the three pooled stage-specific datasets show coordinated differences in relative nuclear representation, transcriptional states, inferred trajectories, and predicted communication profiles. Because the study is cross-sectional and includes one pooled snRNA-Seq library per stage, these observations establish stage-associated patterns rather than direct temporal remodeling or causal involvement in sex reversal.

The identification of 19 neuronal and non-neuronal populations highlights the complexity of the rice field eel OE. Previous zebrafish single-cell studies similarly identified multiple mature and immature OSN populations together with basal, epithelial, and immune compartments [7]. Our results extend this cellular framework to *M. albus*. cOSN nuclei showed lower relative representation in the intersex and male datasets than in the female dataset, whereas basal-cell nuclei also differed in representation, particularly in the male dataset. These descriptive differences should not be equated with changes in absolute cell abundance because nuclear recovery and capture efficiency may vary among cell types and stages.

### 4.2. Basal and Progenitor Compartments Show Stage-Associated Differences

GBC and HBC populations showed notable stage-associated differences in relative nuclear representation. GBC nuclei were relatively prominent in the female and intersex datasets, whereas HBC and ActinHBC nuclei were more highly represented in the male dataset. GBCs and basal progenitor populations contribute to olfactory neuronal maintenance and repair in fishes, whereas HBCs have been extensively characterized as reserve progenitors in mammalian OE [12,13,14]. In mouse OE, single-cell profiling combined with lineage tracing has resolved basal-cell differentiation trajectories [22], while experimental studies have implicated Notch signaling in HBC dormancy and progenitor fate decisions [23,24]. Functional equivalence across vertebrate taxa should therefore be interpreted cautiously.

These observations are compatible with differences in basal/progenitor state across sexual stages, but they do not demonstrate altered regeneration, tissue damage, or lineage behavior. Direct assessment of proliferative or lineage activity will require independent approaches such as EdU/BrdU labeling, lineage tracing, spatial validation, or cell-type-specific molecular assays.

### 4.3. OSN Populations Show Stage-Associated Molecular Heterogeneity

The major OSN populations displayed distinct transcriptional characteristics. Olfactory marker protein is a well-established marker of mature olfactory sensory neurons in vertebrates [25]. Previous zebrafish work has demonstrated molecular specialization among ciliated, microvillous, immature, and crypt neurons [7,10,15]. In the present *M. albus* dataset, *ompa*, *gng8*, and *ora4* showed patterns consistent with the marker-based annotation of cOSNs, mOSNs, and CrOSNs, respectively. In particular, *ora4* was strongly concentrated in the CrOSN cluster, consistent with its established association with crypt neurons in zebrafish [15]. These cross-species similarities support annotation but do not establish identical functions in *M. albus*.

CrOSNs were detected in all three pooled sexual-stage datasets rather than only in the intersex dataset. Their transcriptomic profiles and inferred communication patterns varied among stages, but the present design cannot determine whether these differences represent functional reprogramming, altered precursor dynamics, technical sampling effects, or other biological processes. The appropriate interpretation is therefore stage-associated molecular variation rather than intersex-specific appearance or demonstrated functional remodeling.

### 4.4. CrOSNs Are a Candidate Population for Reproductive Sensory Studies

Crypt neurons are of particular interest because increasing evidence links them to reproductive chemosensation. In zebrafish, *ora4*-expressing neurons form a distinct olfactory population [15]. More recently, Cui et al. demonstrated that GnRH3-associated crypt sensory neurons detect sex-pheromone information and contribute to male courtship and reproductive success [16].

The stage-associated variation of CrOSNs in *M. albus* raises the hypothesis that specialized chemosensory populations may differ as reproductive state changes. However, evidence linking crypt neurons to reproductive chemosensation is currently strongest in zebrafish [15,16], and the present data do not establish the same functional role in *M. albus*. CrOSNs should therefore be regarded as a candidate sensory population for future investigation rather than as a proven trigger or regulator of sex reversal.

### 4.5. Pseudotime Analysis Describes OSN Transcriptional Relationships but Does Not Resolve CrOSN Origin

Pseudotime reconstruction positioned iOSNs within relatively early transcriptional states and placed cOSNs and mOSNs along the principal inferred trajectory. This pattern is compatible with established concepts of immature OSNs as transitional populations during continuous OE neurogenesis [7,8], but the present cross-sectional analysis does not demonstrate actual developmental progression. CrOSNs occupied a comparatively separated region of the inferred trajectory.

Trajectory inference reconstructs transcriptional similarity and possible state relationships from cross-sectional molecular data; it does not constitute direct lineage evidence. The separated position of CrOSNs therefore does not establish an independent developmental lineage. Alternative possibilities include derivation from a distinct precursor population, transient precursor states not captured in the present dataset, or differentiation pathways that are not resolved by this trajectory model. Direct lineage tracing will be required to distinguish among these possibilities.

### 4.6. Cell-Type-Specific Gene-Expression Contrasts Suggest Candidate Functional Differences

The cell-type-resolved analysis identified exploratory stage-associated expression contrasts involving genes related by annotation to neuronal activity, extracellular matrix organization, cell adhesion, tissue repair, metabolic homeostasis, and post-transcriptional regulation. Because one pooled library represented each sexual stage, these contrasts should be interpreted as candidate transcriptional patterns within the present dataset rather than as independently replicated evidence of pathway-level reprogramming.

Several recurrent genes have functions that may be relevant to OE biology. *fosb* encodes a Fos-family component of the AP-1 transcription factor complex, whereas *gad1b* encodes a glutamate decarboxylase involved in GABA synthesis. Their stage-associated expression patterns are compatible with differences in neuronal or neuroepithelial transcriptional state, but they do not establish a direct role in sex reversal.

Other candidate genes were associated by conserved annotation with extracellular matrix organization and cell–matrix interactions. *mmp9* participates in extracellular matrix turnover, *plod1a* is associated with collagen modification, and *itga5* encodes the alpha-5 integrin subunit involved in fibronectin-mediated adhesion and signaling [26]. These gene-level observations may indicate differences in structural or adhesive programs, but pathway-level conclusions require independent replication and enrichment analysis.

*areg* encodes amphiregulin, an epidermal growth factor receptor ligand with established roles in epithelial homeostasis, inflammation, and tissue repair [27]. Its stage-associated expression, together with differences in HBC and ActinHBC nuclear representation, is compatible with variation in epithelial state; however, the present data do not demonstrate altered regenerative activity or a causal role for amphiregulin.

Additional genes suggest candidate metabolic differences. *slc31a2* encodes a copper transporter involved in cellular copper handling [28], whereas *cdo1* and *csad* participate in cysteine catabolism and the cysteine-sulfinate pathway. apoeb is associated with lipid transport, and *piwil1* is involved in RNA-regulatory processes. Because these interpretations rely largely on conserved annotations and exploratory contrasts, their specific roles in the rice field eel OE require experimental verification.

Taken together, these gene-level contrasts nominate candidate processes for future study but do not establish coordinated pathway-level remodeling. Independent biological replication, replicate-aware differential-expression analysis, and pathway enrichment would be required to test whether the suggested neural, structural, metabolic, or regulatory programs differ reproducibly among sexual stages.

### 4.7. Predicted Cell–Cell Communication Profiles Are Exploratory

CellChat analysis produced different nominal network sizes and predicted interaction strengths among the three datasets. Male OE contained 1517 inferred interactions, compared with 1114 in female and 1202 in intersex OE, whereas the corresponding predicted overall interaction strength was lower in the male dataset. Because these values were generated using a human ligand–receptor database applied to *M. albus*, they should be treated as dataset-specific computational descriptors rather than quantitative evidence of altered in vivo communication.

The apparent differences in predicted signaling profiles may be useful for prioritizing candidate ligand–receptor axes, but terms such as diversification or redistribution of signaling cannot be established directly from the present analysis. Species-specific orthology, receptor specificity, and independent in vivo validation will be required before functional conclusions can be drawn.

### 4.8. Immune and Stromal Populations Contribute to the OE Cellular Landscape

Macrophages, dendritic cells, B cells, CD8-like cells, thrombocytes, mesenchymal cells, and endothelial cells were identified in the rice field eel OE dataset, demonstrating a substantial non-neuronal component. Immune populations have also been identified in zebrafish OE single-cell datasets [7]. In the present study, B-cell and other immune-cell nuclei showed stage-associated differences in relative representation. However, these observations are descriptive and may reflect both biological variation and differences in nuclear recovery; they do not by themselves establish neuroimmune remodeling.

Future analyses with independent biological replication and spatial validation could determine whether immune, stromal, endothelial, and progenitor populations show reproducible changes and whether specific ligand–receptor relationships contribute to OE physiology during sexual transition.

### 4.9. Potential Biological Relevance of OE Differences During Sex Reversal

Natural sex reversal involves changes in reproductive role as well as gonadal phenotype. Because olfaction provides information relevant to mate recognition, reproductive status, social interaction, and environmental conditions, stage-associated OE differences are biologically plausible. The present data are consistent with this possibility at the cellular and transcriptomic levels, but they do not demonstrate that OE variation contributes causally to the sex-reversal process.

Evidence for functional links between olfactory and reproductive systems comes primarily from other teleost models, including zebrafish studies of olfactory GnRH3-associated crypt sensory neurons [16] and GnRH3 neuronal heterogeneity in the olfactory region [17]. These findings provide comparative context but should not be interpreted as demonstrating an equivalent OE-to-reproductive endocrine mechanism in *M. albus*.

### 4.10. Limitations and Future Perspectives

Several limitations should be considered. First, OE tissues from five individuals within each sexual stage were pooled before nuclei isolation, yielding one snRNA-Seq library per stage. Consequently, individual-to-individual biological variation cannot be estimated, individual nuclei cannot be treated as independent biological replicates, library identity is fully confounded with sexual stage, and cell-composition or gene-expression contrasts among stages should be interpreted as exploratory and descriptive rather than as statistically replicated population-level differences. Second, snRNA-Seq nuclear recovery and capture efficiency may differ among cell populations and stages; relative nuclear representation therefore may not correspond to absolute histological cell abundance. Third, the market origin of the animals means that precise prior environmental history and genetic background were unavailable. Fourth, pseudotime analysis reconstructs transcriptional relationships but cannot establish definitive lineage relationships, particularly for CrOSNs. Because each stage corresponds to a different library, library effects may also contribute to inferred trajectory positions and branch structure. Fifth, cell-type annotations are marker-based and several specialized populations require spatial or protein-level validation. Sixth, CellChat analysis used CellChatDB.human because a species-specific *M. albus* ligand–receptor database is unavailable; therefore, inferred interaction counts and strengths are nominal predictions and cross-species mapping may introduce biologically mismatched interactions. Seventh, snRNA-Seq does not preserve the spatial organization of the OE, so apparent differences in zone-associated populations cannot be distinguished from changes in epithelial boundaries or nuclear recovery without spatial validation. Finally, Figure 5 visualizes marker-gene expression within the same snRNA-Seq dataset and does not constitute independent RT-PCR, RT-qPCR, spatial, or protein-level validation. Incomplete genome annotation in *M. albus* further limits functional interpretation of some differentially expressed loci because several transcripts remain annotated only by LOC/accession identifiers without validated gene symbols or full gene names.

Future studies should prioritize independently replicated sequencing or replicate-aware validation across individual animals, together with spatial approaches such as RNAscope, in situ hybridization, immunohistochemistry, or spatial transcriptomics. Additional work combining sexual-stage analysis with circulating steroid measurements, exposure to reproductive chemical cues, calcium imaging or electrophysiological recording, neural tracing, and manipulation of GnRH-associated pathways could then test the functional significance of candidate OE populations and signaling relationships.

## 5. Conclusions

The present snRNA-Seq dataset identifies stage-associated differences in the relative nuclear representation and transcriptional characteristics of multiple olfactory epithelial populations across female, intersex, and male *M. albus*. Nineteen major cell populations were annotated based on transcriptomic profiles, including sensory neuronal, basal/progenitor, epithelial, immune, stromal, and vascular populations. The datasets also showed differences in inferred OSN transcriptional relationships, exploratory cell-type-specific gene-expression contrasts, and predicted ligand–receptor communication profiles. Because one pooled sequencing library was generated per sexual stage, these observations should be considered exploratory and require validation using independently replicated and spatially resolved approaches.

CrOSNs were detected in all three sexual-stage datasets rather than being restricted to the intersex stage. Their *ora4* expression, distinct inferred trajectory position, and stage-associated transcriptomic patterns highlight CrOSNs as a candidate population for future study. The present findings provide an initial transcriptomic resource for investigating peripheral olfactory variation associated with natural sex reversal in *M. albus*, without establishing a causal role of OE changes in the sex-reversal process.

## Data Availability

The raw snRNA-Seq data have been deposited in CNCB (https://ngdc.cncb.ac.cn/gsub/submit/bioproject/subPRO108990/overview; accessed on 23 September 2026) under accession number PRJCA075250.

## Supplementary Materials

The following supporting information can be downloaded at: https://www.mdpi.com/article/10.3390/ani16203233/s1, Table S1: Sequencing and quality-control metrics for the three snRNA-seq libraries; Table S2: Numbers and percentages of nuclei assigned to each cell type in the female, intersex, and male libraries; Table S3: Marker genes for cell type definition; Figure S1: Representative gonadal histology used for sexual-stage classification in *Monopterus albus*. H&E-stained gonadal sections from female, intersex, and male individuals are shown from left to right. Female gonads were characterized by predominantly ovarian tissue, intersex gonads by simultaneous ovarian and testicular characteristics, and male gonads by predominantly testicular tissue. These histological features, together with gonadal morphology, were used to assign individuals to the three sexual stages; Figure S2: Exploratory prediction of cell-cell communication profiles across sexual stages. (A) Predicted outgoing and incoming communication strengths of annotated popula-tions. Bubble size represents predicted interaction number. (B) Pairwise interaction heatmaps. All values were computationally inferred using CellChatDB.human and should be interpreted quali-tatively rather than as experimentally validated communication events.

## Abbreviations

ActinHBCs, activated horizontal basal cells; Bcell, B cells; cOSNs, ciliated olfactory sensory neurons; CrOSNs, crypt olfactory sensory neurons; DEG, differentially expressed gene; GBCs, globose basal cells; HBCs, horizontal basal cells; iOSNs, immature olfactory sensory neurons; mOSNs, microvillous olfactory sensory neurons; OE, olfactory epithelium; OSN, olfactory sensory neuron; PCA, principal component analysis; snRNA-Seq, single-nucleus RNA sequencing; UMAP, Uniform Manifold Approximation and Projection; UMI, unique molecular identifier.
