# Supplementary material for: Single-Nucleus Transcriptomics Identifies Stage-Associated Differences in the Olfactory Epithelium During Natural Sex Reversal in Monopterus albus
Source: Animals (Basel). 2026 Oct 14;16(20):3233. doi: 10.3390/ani16203233 (PMC13700653; doi:10.3390/ani16203233)
Supplement: Supplementary file 1 [file animals-16-03233-s001.zip › animals-4552338-supplementary/Supplementary Figure.pdf]

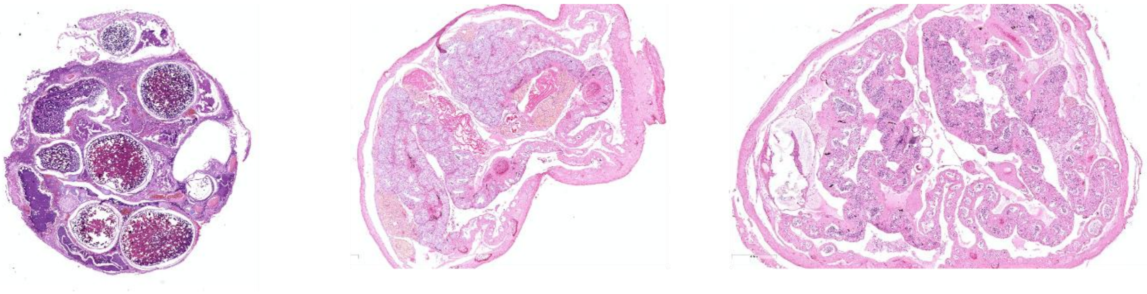

**Female** **Intersex** **Male**

**Figure S1. Representative gonadal histology used for sexual-stage classification in *Monopterus albus*.** H&E-stained gonadal sections from female, intersex, and male individuals are shown from left to right. Female gonads were characterized by predominantly ovarian tissue, intersex gonads by simultaneous ovarian and testicular characteristics, and male gonads by predominantly testicular tissue. These histological features, together with gonadal morphology, were used to assign individuals to the three sexual stages.

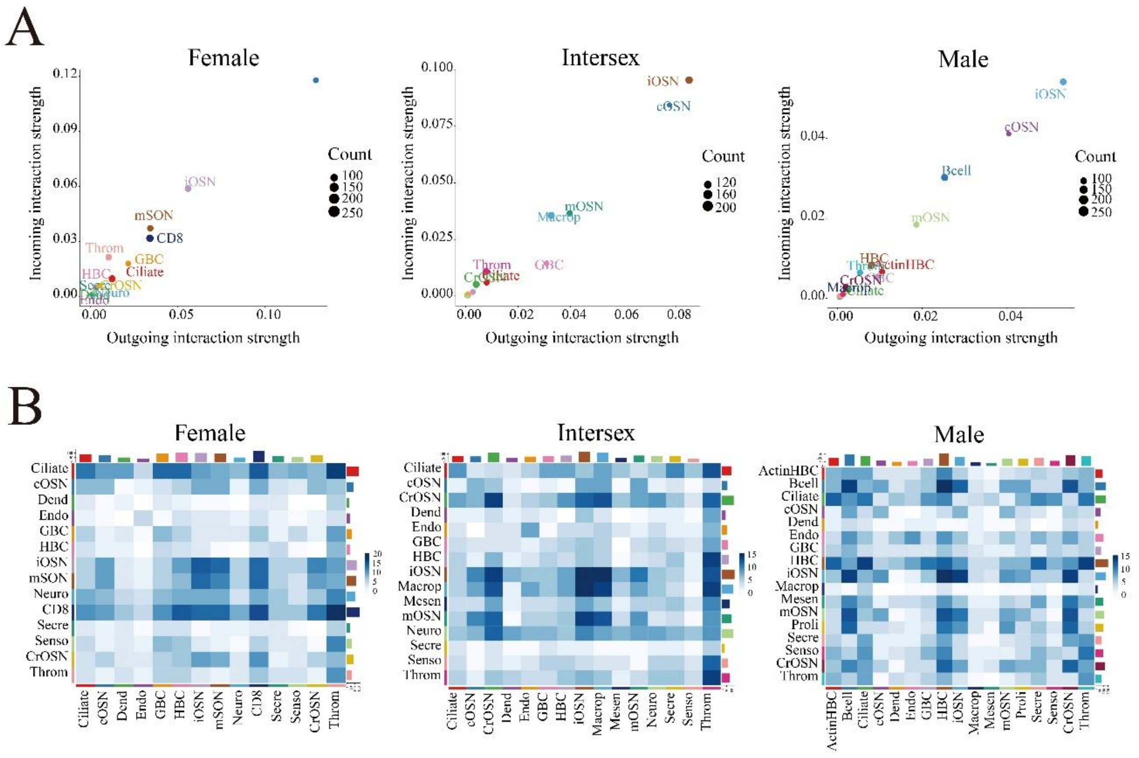


**Figure S2. Exploratory prediction of cell-cell communication profiles across sexual stages.** (A) Predicted outgoing and incoming communication strengths of annotated popula-tions. Bubble size represents predicted interaction number. (B) Pairwise interaction heatmaps. All values were computationally inferred using CellChatDB.human and should be interpreted quali-tatively rather than as experimentally validated communication events.
